# A high-dimensional, multi-transceiver channel state information dataset for enhanced human activity recognition

**DOI:** 10.1016/j.dib.2024.110673

**Published:** 2024-06-25

**Authors:** Wei Ern Wong, An Hong Wong, Wei Qi Peh, Chee Keong Tan

**Affiliations:** Monash University, Jalan Lagoon Selatan, Bandar Sunway, 47500 Subang Jaya, Selangor, Malaysia

**Keywords:** Human activity recognition, Wi-Fi IEEE 802.11n, Channel statement information (CSI), ESP32 transceivers, Spatial diversity, High dimensionality

## Abstract

Human Activity Recognition (HAR) has emerged as a critical research area due to its extensive applications in various real-world domains. Numerous CSI-based datasets have been established to support the development and evaluation of advanced HAR algorithms. However, existing CSI-based HAR datasets are frequently limited by a dearth of complexity and diversity in the activities represented, hindering the design of robust HAR models. These limitations typically manifest as a narrow focus on a limited range of activities or the exclusion of factors influencing real-world CSI measurements. Consequently, the scarcity of diverse training data can impede the development of efficient HAR systems. To address the limitations of existing datasets, this paper introduces a novel dataset that captures spatial diversity through multiple transceiver orientations over a high dimensional space encompassing a large number of subcarriers. The dataset incorporates a wider range of real-world factors including extensive activity range, a spectrum of human movements (encompassing both micro-and macro-movements), variations in body composition, and diverse environmental conditions (noise and interference). The experiment is performed in a controlled laboratory environment with dimensions of 5 m (width) × 8 m (length) × 3 m (height) to capture CSI measurements for various human activities. Four ESP32-S3-DevKitC-1 devices, configured as transceiver pairs with unique Media Access Control (MAC) addresses, collect CSI data according to the Wi-Fi IEEE 802.11n standard. Mounted on tripods at a height of 1.5 m, the transmitter devices (powered by external power banks) positioned at north and east send multiple Wi-Fi beacons to their respective receivers (connected to laptops via USB for data collection) located at south and west. To capture multi-perspective CSI data, all six participants sequentially performed designated activities while standing in the centre of the tripod arrangement for 5 s per sample. The system collected approximately 300–450 packets per sample for approximately 1200 samples per activity, capturing CSI information across the 166 subcarriers employed in the Wi-Fi IEEE 802.11n standard. By leveraging the richness of this dataset, HAR researchers can develop more robust and generalizable CSI-based HAR models. Compared to traditional HAR approaches, these CSI-based models hold the promise of significantly enhanced accuracy and robustness when deployed in real-world scenarios. This stems from their ability to capture the nuanced dynamics of human movement through the analysis of wireless channel characteristic from different spatial variations (utilizing two-diagonal ESP32 transceivers configuration) with higher degree of dimensionality (166 subcarriers).

Specifications Table

**Table:** 

Subject	Applied Machine Learning
Specific subject area	Human Activity Recognition (HAR) Using Channel State Information (CSI) via Wi-Fi Sensing
Type of data	Table (.csv)
Data collection	In a controlled environment, CSI data for 10 human macro- and micro-movement were collected using 4 ESP32 1 transceivers (2 m apart), mounted on 1.5 m tripods operating under Wi-Fi IEEE 802.11n on channel 11. Each pair had unique MACs to ensure spatial diversity. The transmitters powered externally faced inwards from north and east towards the receivers (connected to laptops) at south and west, with participants in the centre of the designated area. To account for body variations, 6 individuals performed activities sequentially for 5 s (1 sample). The system collected 300–450 packets/sample across 166 subcarriers, and data was converted to CSV files with timestamps for traceability.
Data source location	Institution: Monash University City/Town/Region: Selangor, Subang Jaya, 47,500 Country: Malaysia Latitude and longitude (and GPS coordinates) for collected samples/data: 3.0650° N, 101.6009° E
Data accessibility	Repository name: SHD-HAR-Dataset Data identification number: doi.org/10.5281/zenodo.11201414 Direct URL to data: https://zenodo.org/records/11201414
Related research article	None

## 1 Value of the Data

• The dataset offers a unique contribution to the field of CSI-based HAR. It represents, to the best of our knowledge, the first publicly available dataset capturing CSI measurements from two independent transceivers positioned in a diagonal network. This diagonal transceiver configuration plays a crucial role in capturing richer and more informative data for HAR. Unlike conventional setups with co-located transceivers, our diagonal arrangement provides spatially diverse data. This means that the signal travels across different paths in the environment due to the physical separation of the transceivers. This diversity offers the potential to capture different perspectives on human activities within the same space. Imagine a person walking across a room. A co-located transceiver setup might primarily capture data from a single direction. However, the diagonal configuration can collect signals that bounce off walls or furniture, providing additional information about the activity from various angles. This can be particularly beneficial for recognizing complex activities or differentiating between similar movements. By capturing a comprehensive range of spatial information, the diagonal transceiver configuration allows for the development of more robust and accurate CSI-based HAR models. We believe this dataset will be a valuable resource for researchers in this field. The dataset distinguishes itself from existing CSI-based HAR datasets by incorporating a broader spectrum of human activities. Unlike prior resources, which often focus on macro-movement performed by a single individual, our dataset encompasses ten micro- and macro-movements executed by six participants with varying body compositions. This rich tapestry of data facilitates the exploration of the intricate interplay between human body composition, multi-perspective CSI measurements captured from two transceivers placed diagonally, and diverse activity types. This comprehensive approach empowers researchers to develop more sophisticated and generalizable HAR models.
• The dataset provides a rich resource for CSI-based HAR research, encompassing 300–450 packets per sample (for approximately 1200 samples for each activity) for various human activities (macro- and micro-movements). The richness extends to the inclusion of data collected across different time periods, enabling researchers to analyze the impact of temporal variations in interference and noise on CSI measurements.
• The dataset stands out for its unparalleled granularity in capturing CSI by increasing the dimensionality compared to the existing CSI-based datasets. It records data over a high dimensional space across a remarkable 166 subcarriers, the most extensive among existing CSI-based HAR datasets. This comprehensive subcarrier capture offers significant advantages for activity recognition. By incorporating a wider range of frequency variations, the dataset provides a richer and more nuanced representation of the signal propagation patterns affected by human movement. This enhanced detail can lead to improved accuracy and robustness in machine learning models trained for HAR tasks.
• The dataset empowers researchers to design more advanced HAR systems by facilitating the exploration of multi-dimensional CSI data under diverse real-world conditions. These conditions encompass, but are not limited to, noisy CSI measurements, a comprehensive range of human activities (both micro- and macro-movements), variations in body composition among participants, different subcarriers’ characteristics, and the impact of different environmental factors (interference) on signal propagation.
• Deep learning-based HAR models trained on this rich CSI datasets transcend the limitations of smart homes and hold immense potential for real-world applications across diverse domains. These models, with their ability to decipher human activities through CSI data, offer valuable insights for applications like monitoring elderly individuals for falls, tracking patient progress in physical therapy, identifying unsafe postures in hazardous workplaces, creating intuitive gesture controls for video games, optimizing building automation based on occupancy, and understanding customer behaviour in retail stores through browsing patterns. By enabling such functionalities, these HAR models have the potential to revolutionize healthcare, safety, entertainment, energy efficiency, and marketing strategies.

## 2 Background

The dataset explores the influence of human activity on CSI measurements by leveraging a unique collection captured within a controlled environment using two sets of ESP32 devices configured as transceiver pairs, arranged in a diagonally opposing configuration. Driven by the goal of improved HAR through CSI analysis, the experiment gathered rich CSI data offering valuable insights for researchers in this field. While traditional research relies on single transceiver setups, this dataset leverages two transceivers, potentially capturing a wider range of data due to their diagonal spatial configuration. Analysing data from this multiple spatiality can lead to more robust and comprehensive HAR models. Expanding upon existing CSI-based HAR dataset's focus on macro-movements, this collection incorporates both micro- and macro-movements from multiple individuals, resulting in a richer data resource that facilitates the training and evaluation of HAR models with enhanced sensitivity to subtle human actions. Another compelling motivation of this dataset is to facilitates exploration of how body composition and ESP32 configuration interact with CSI measurements. The dataset incorporating CSI information from six participants of diverse body types, captured over a high dimensionality across 166 subcarriers, provides a valuable platform for analysing the influence of subcarrier characteristics on the acquired CSI measurements. This dataset facilitates the development of HAR systems with demonstrably improved reliability and accuracy in real-world applications.

## 3 Data Description

The field of HAR has witnessed significant growth in recent years, driven by its potential to revolutionize various sectors. From healthcare applications like monitoring patient activity and fall detection to fitness tracking and smart environment automation, HAR offers a versatile technology with far-reaching implications [1]. Deep learning has become a prominent tool for HAR tasks. Its ability to extract intricate patterns from large datasets enables deep learning models to achieve impressive accuracy in classifying diverse human activities [2]. However, the success of these models is inherently tied to the quality and diversity of the training data they are exposed to. This is where CSI-based datasets emerge as a vital resource.

CSI data, derived from the analysis of wireless signals, presents a unique and promising avenue for HAR. Unlike traditional sensor modalities, such as body-worn accelerometers, gyroscopes and magnetometers, CSI data offers several advantages. It is inherently non-invasive, requiring no specific user devices, and privacy-preserving, as it doesn't rely on identifying individuals. Additionally, CSI data captures subtle variations in the wireless channel induced by human movement. This rich information provides a valuable foundation for deep learning models to accurately recognize and classify a wide range of human activities.

While the field of CSI-based HAR has seen a rise in the number of available datasets (as summarized in Table 1), there exist limitations that hinder their effectiveness and overall usability. Here, we address these shortcomings and introduce a novel dataset designed to overcome these limitations and contribute to the advancement of CSI-based HAR research. Current datasets often suffer from several limitations. One key constraint is the reliance on a single transceiver configuration. This restricted setup captures only a limited spatial representation of movement, potentially failing to capture the nuances of human activity. Additionally, some datasets utilize a limited subset of subcarriers, resulting in lower data dimensionality. This can have a negative impact on the performance of deep learning models, which often rely on rich data features for accurate recognition. Furthermore, the range of human activities represented in existing datasets may be narrow. Many datasets focus only on larger, more pronounced movements (macro-movements) and neglect the potential value of including subtle micro-movements in activity recognition tasks. Finally, the influence of body composition on how individuals interact with the wireless channel is often disregarded. This lack of consideration can limit the generalizability of HAR models, as individuals with varying body types might exhibit distinct CSI variations during activities. Our dataset addresses these shortcomings through a comprehensive data collection strategy. We incorporate multi-transceiver configurations to capture a richer spatial representation of movement. Additionally, we utilize a broader range of subcarriers, providing higher data dimensionality for deep learning models to leverage. The dataset encompasses a diverse spectrum of human activities, including both macro-movements and subtle micro-movements, offering a more comprehensive view of human behaviours. Finally, we consider the role of body composition by including participants with varying body types. This multifaceted approach ensures the dataset's suitability for developing more accurate, robust, and generalizable HAR models using CSI data. By addressing these limitations and incorporating these novel elements, our dataset offers valuable contributions to the field of CSI-based HAR research. It provides a richer and more diverse foundation for researchers developing and evaluating HAR models that can effectively recognize a wider range of human activities with greater accuracy and generalizability.

**Table 1:** CSI-based dataset for HAR models.

Datasets	Devices	Spatiality	Human activities	Dimensionality	Details	Other Factors
P. M. Fard, et al. [3]	Raspberry Pi	1 path	Macro-movement	50 subcarriers	1 testbed 3 individuals 7 activities 20 samples/activity	N.A.
S. Yousefi, et al. [4]	Intel NIC 5300	1 path	Macro-movement	30 subcarriers	1 testbed 6 individuals 6 activities 20 samples/activity	N.A.
S. Arshad, et al. [5]	Transmitter: Linksys EA4500 Dual Band Receiver: laptop (Sony Vgn series) with an Intel WiFi NIC 5300 network adapter.	1 path	Macro-movement	30 subcarriers	1 testbed 12 individuals 3 activities 20 samples/activity	N.A.
Z. Andrii et al. [6]	Linux 802.11n CSI Tool	1 path	Macro-movement	114 subcarriers	3 testbeds 1 individual 7 activities No information on sample size	N.A.
Our Dataset (SHD-HAR) [7]	ESP32-S3-DevKitC-1	2 paths (diagonal)	Micro- and macro-movement	166 subcarriers	1 testbed 6 individuals 10 activities ≈1200 samples/activity	Noise and interference

**Fig. 1:**
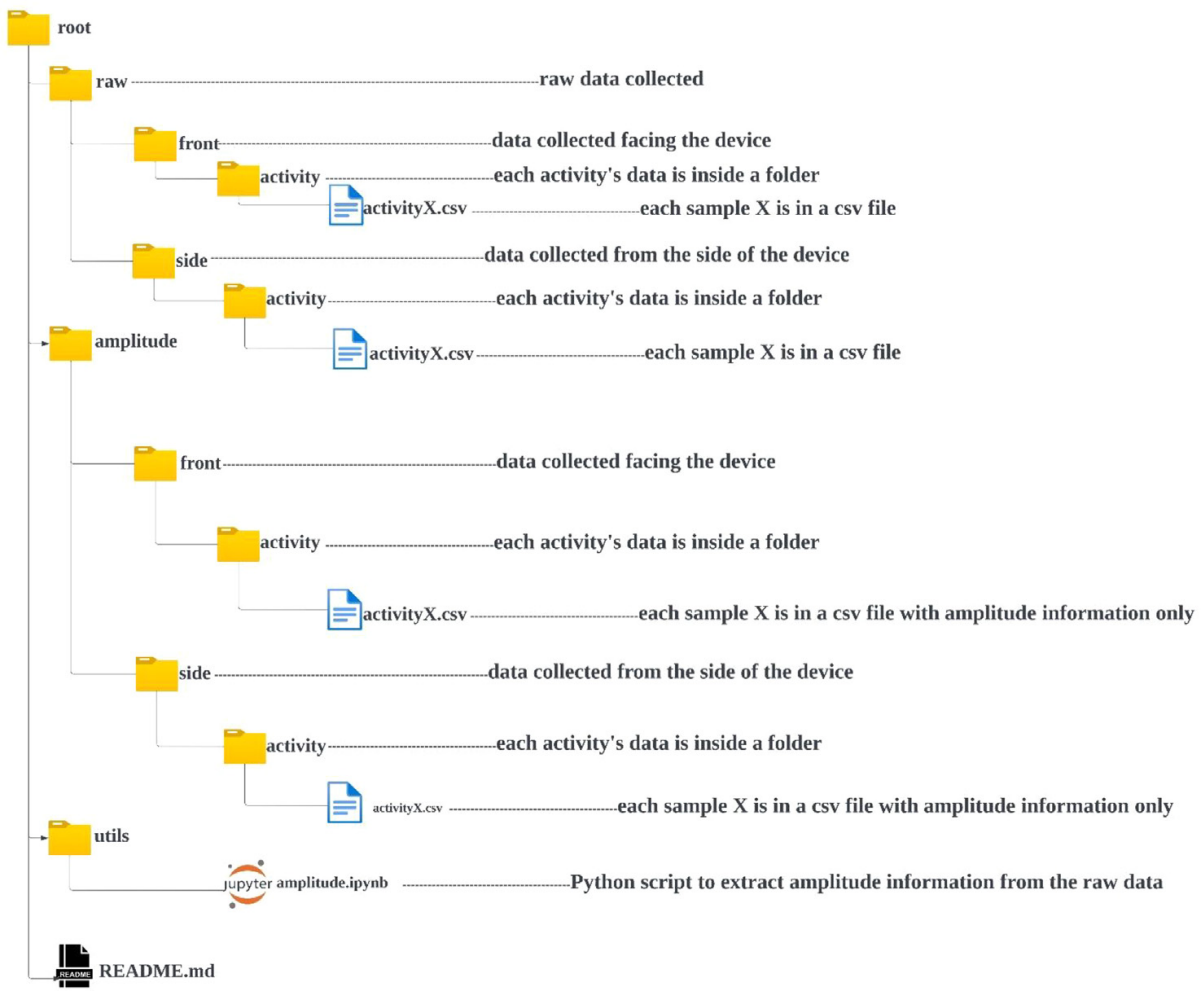
SHD-HAR repository structure.

This article describes a dataset and its associated files stored in a specific repository. Fig. 1 depicts the structure of this repository. The repository on SHD-HAR-Dataset can be accessed via https://zenodo.org/records/11201414 [7]. The dataset itself is stored within a directory named “SHD-HAR-Dataset-main” inside the repository. This directory contains two subdirectories: “raw” and “amplitude.” Both “raw” and “amplitude” directories are further divided into “front” and “side” folders. Data in the “front” folder corresponds to recordings where the participant was facing the ESP32 transceivers (from north to south) performing an activity. Conversely, the “side” folder stores data collected when the participant was positioned sideways relative to another ESP32 transceivers (east to west). Each of the “front” and “side” folders contains ten subdirectories, each named after a specific activity performed by the participant during data collection. Each activity subdirectory holds a single CSV file for each sample collected during that activity. In essence, each CSV file represents a single instance of the corresponding activity. It's important to note that the samples are synchronized between the “front” and “side” folders. This is because both devices collected data simultaneously for a particular activity. To summarize, the file “activityX.csv” in the “front” folder was collected at the same time as the corresponding “activityX.csv” file in the “side” folder, where “X” represents a unique activity identifier for each sample. The raw data for each activity sample comprises 300–450 packets. Each packet contains information associated with 25 distinct fields, resulting in a total of 300–450 × 25 data entries per activity stored within the “.csv” file. For brevity, Table 2 describes the 25 fields of each csv file in the “raw” directory.

**Table 2:** Fields contained in each collected raw sample [9].

Column Name	Column Description
**Type**	CSI_DATA
**Id**	Identifier for the data entry.
**Mac**	Media Access Control (MAC) address of source.
**Rssi**	Received signal strength indicator in dBm, indicating the strength of signal power received.
**Rate**	PHY rate encoding of the packet. Only valid for non-HT (11bg). Also indicates Data FEC coding and modulation aka "MCS"
**sig_mod**	Signal mode. 0: non-HT (11bg) packet; 1: HT (11n) packet; 3: VHT (11ac) packet
**Mcs**	Modulation Coding Scheme. If is HT (11n) packet, shows the modulation, range from 0 to 76 (MSC0 ∼ MCS76).
**Bandwidth**	Radio channel bandwidth 0: 20 MHz; 1: 40 MHz. Bigger bandwidth, more symbols can be transmitted per Hz and bigger data rate. Also means more subcarriers.
**Smoothing**	No info
**not_sounding**	No info
**Aggregation**	Aggregation. 0: MPDU packet; 1: AMPDU packet by default, no aggregation
**Stbc**	Space Time Block Code (STBC). 0: non STBC packet; 1: STBC packet by default, STBC is not used. MIMO is used
**fec_coding**	Forward Error Coding. Flag is set for 11n packets which are LDPC (Low Density Parity Check)
**Sgi**	Short Guide Interval (SGI). 0: Long GI; 1: Short GI
**noise_floor**	The Noise Floor in dBm. It is the signal created from adding up all the unwanted signals within a measurement system. The noise floor consists of noise from a number of sources which includes thermal noise, atmospheric noise and noise from components used to make the measurement system. It is dependent on individual receiver.
**ampdu_cnt**	A-MPDU aggregation is to join Multiple MPDU sub frames with a single leading PHY header. No much info provided
**Channel**	WiFi channel with size 20 MHz. For 11b/g/n in 2.4 GHz there are 11 channels (1 to 11)
**secondary_channel**	Secondary channel on which this packet is received. 0: none; 1: above; 2: below (see Table X for subcarriers indexing)
**local_timestamp**	The local time when this packet is received. It is precise only if modem sleep or light sleep is not enabled. unit: microsecond
**Ant**	Antenna number from which this packet is received. 0: WiFi antenna 0; 1: WiFi antenna 1
**sig_len**	Signal length. length of packet including Frame Check Sequence (FCS)
**rx_state**	state of the packet. 0: no error; others: error numbers which are not public
**Len**	Length of CSI data in bytes, total of 384 bytes in our dataset sample
**first_word**	the first four bytes of the CSI data is invalid or not. 1=valid
**Data**	CSI data corresponding to each Long Training Field (LTF) type is stored in a buffer starting from the buf field. Each item is stored as two bytes: imaginary part followed by real part. The order of each item is the same as the sub-carrier in the table. The order of LTF is: LLTF, HT-LTF, STBC—HT-LTF

The dataset repository extends beyond the raw data, offering a Python package named *utils* for user convenience. This package includes a Jupyter Notebook (amplitude.ipynb) that facilitates data preprocessing by converting raw data into amplitude information. Additionally, a README.md file provides a concise overview of the repository's structure and content. The functions used in amplitude.ipynb are summarized in Table 3.

**Table 3:** Summary of functions used for amplitude.ipynb.

Functions	Description
Class ESP32	Parse ESP32 Wi-Fi Channel State Information (CSI) obtained using ESP32 CSI Toolkit by Hernandez and Bulut.
**Function inside this class:**	
read_file	Read RAW CSI file (.csv) using Pandas and return a Pandas data frame
seek_file	Seek RAW CSI file
filter_by_sig_mode	Filter CSI data by signal mode Parameters: sig_mode (int): 0: Non - High Throughput Signals (non-HT) 1: High Throughput Signals (HT)
get_csi	Read CSI string as Numpy array - The CSI data collected by ESP32 contains channel frequency responses (CFR) represented by two signed bytes (imaginary, real) for each subcarriers index - The length (bytes) of the CSI sequency depends on the CFR type CFR consist of legacy long training field (LLTF), high-throughput LTF (HT-LTF), and space- time block code HT-LTF (STBC-HT-LTF)
remove_null_subcarriers	Remove NULL subcarriers from CSI
get_amplitude_from_csi	Calculate the Amplitude (or Magnitude) from CSI
get_phase_from_csi	Calculate the Phase (or Magnitude) from CSI
amplitude_plot	Plotting function for visualising subcarrier amplitude per packet. Parameters: amp: csi amplitude array start_stamp: start plot from which packet num_packet: plot how many packets plot_name: name of activity being plotted
csv_amplitude	csv_amplitude Function to convert directory of raw csv files to amplitude csv files Parameters: directory: directory containing raw csv files
directory_to_heatmap	Function to convert amplitude files to heatmap jpg Parameters: directory: amplitude file directory dim: length and width of jpg

**Fig. 2:**
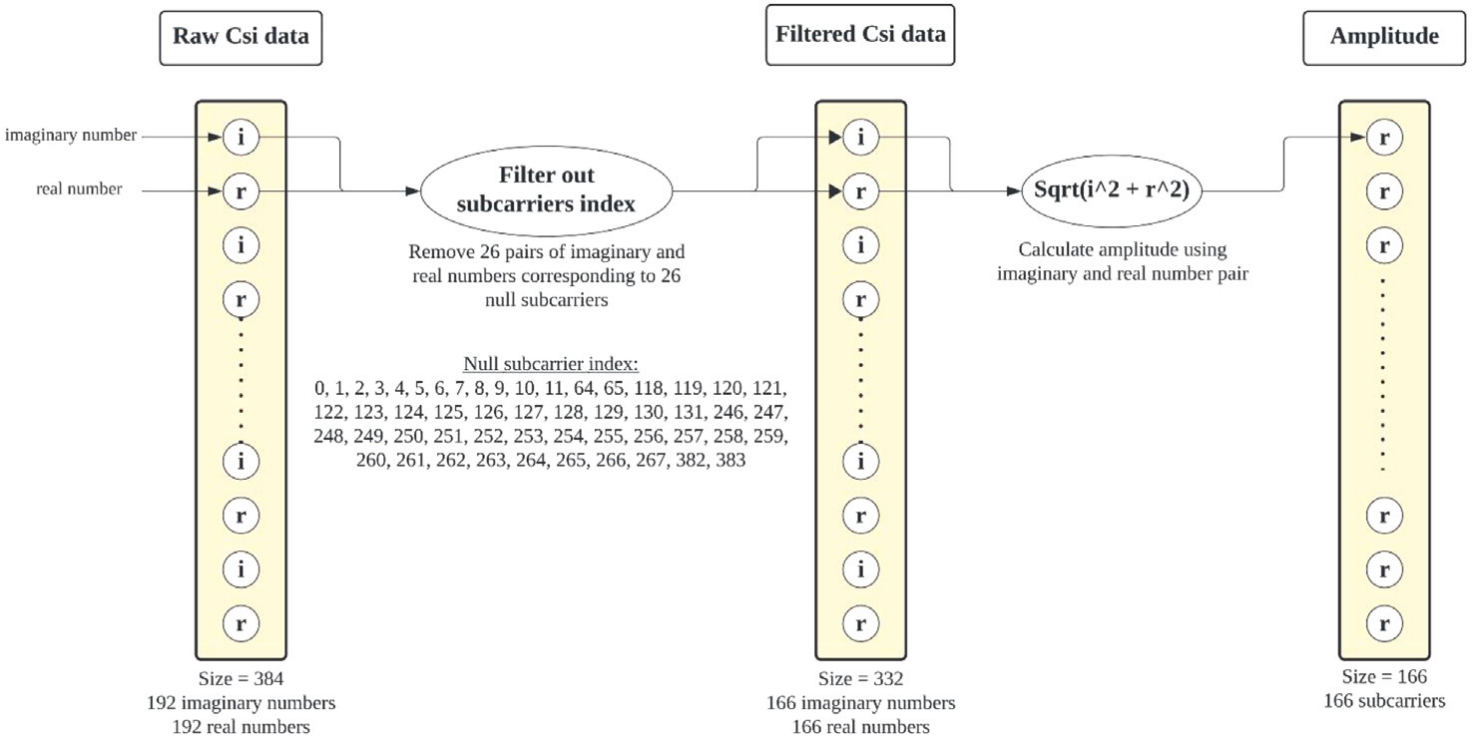
Preprocessing of raw CSI data for amplitude extraction.

The dataset package incorporates a preprocessing step to convert raw CSI data into amplitude data suitable for further analysis. This process (as illustrated in Fig. 2) involves two main steps: 1) subcarrier filtering and 2) subcarrier reduction. Raw CSI data typically includes information from a large number of subcarriers, which are frequency bins used for signal transmission. Some subcarriers may contain null values, potentially due to missing data or specific channel conditions. The filtering process removes these subcarriers with null values to ensure data quality and consistency. The dataset optimizes processing by filtering null subcarriers and reducing dimensionality from 192 to 166, preserving core information for analysis. Once the raw CSI data undergoes subcarrier filtering and reduction, the remaining subcarriers are processed to extract the amplitude information. The CSV files within the amplitude directory contain data entries for each sample. These entries are structured as a matrix with dimensions of 300–450 rows and 166 columns. This typically involves calculating the magnitude of the complex-valued data for each subcarrier. The magnitude represents the signal strength or amplitude, which can be used for various tasks like HAR or channel modeling.

**Fig. 3:**
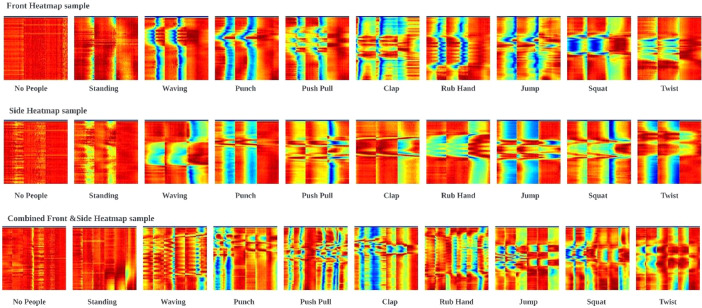
Sample heatmaps for 10 human activities for different orientations (i.e., front, side and combined front & side) including micro and macho activities.

To validate the datasetʼs usability for HAR, a deep learning model based on a Convolutional Neural Network (CNN) architecture can be employed [8]. This model leverages a combination of feature extraction and data preprocessing techniques. The feature extraction technique utilizes Discrete Wavelet Transform while low-pass filtering serves as the data preprocessing technique. Prior to feeding the data into the CNN model, the raw data is preprocessed to retain only amplitude information. This processed data is then visualized as heatmaps for each sample, as illustrated in Fig. 3. These heatmaps depict various activities captured from different data collection orientations, i.e., front, side and combined front & side. Validation of the datasets using an unoptimized baseline deep learning model yields the accuracy results outlined in Table 4. This validation confirms the usability of the dataset for recognizing various human activities across various device orientations.

**Table 4:** Validation accuracy of CNN model for HAR on the proposed dataset.

Orientation	Subsets of activities	Validation accuracy
Front	Baseline + Macro	92.34
	Micro	47.30
	All	61.49

Side	Baseline + Macro	88.87
	Micro	55.00
	Baseline + Macro	60.89

Combined front & side	Baseline + Macro	82.46
	Micro	49.97
	Baseline + Macro	63.66

## 4 Experimental Design, Materials and Methods

### 4.1 Subjects and activities

Six researchers with different heights and weights participated in the data collection process. Table 5 presents details on the subjects, including gender, age, height (cm), and weight (kg). Our study prioritizes transparency and inclusivity in participant selection. To minimize potential bias based on physical characteristics, we opted for an open and voluntary recruitment process. Compared to single-participant studies often found in existing datasets, voluntary recruitment allowed us to gather data from a significantly larger and more diverse pool of individuals with heights (156–177 cm) and weights (38–56 kg) as shown in Table 5. This enriches the dataset by incorporating a wider range of body types and potentially capturing a broader spectrum of variations in the CSI measurements. These variations could be influenced by factors like height, weight, and overall body composition. By including a wider range of participants, the dataset has the potential to be more generalizable to real-world scenarios with diverse populations. Unlike studies with pre-determined physical criteria, this method avoids any concerns about potential bias in choosing participants. This builds trust in the study's methodology and ensures that the results are a reflection of the data collected from a wider range of individuals. While voluntary recruitment offers clear advantages, it also introduces a limitation in terms of participant diversity with respect to body composition such as representation of individuals with extreme body mass indexes (BMIs). Since the selection of participants is not pre-determined, the current participant pool does not encompass the full spectrum of BMIs. Future studies could explore recruitment strategies that target a more diverse range of body compositions, including extreme BMIs in the participant pool.

**Table 5:** Subject demographics: gender, age, height, and weight.

Test Subject No	Gender	Age	Height (cm)	Weight (kg)
1	Male	22	174	55
2	Male	21	163	56
3	Male	21	177	50
4	Female	21	154	38
5	Female	21	162	45
6	Female	22	156	48

The activities were categorized into three main groups: baseline activities, macro-movement (big body movement), and micro-movement (hand gestures). Each category encompassed a specific set of actions, resulting in a total of 10 activities proposed for data collection. Baseline activities included an empty experimental environment and a participant standing still between the data collection devices (labeled with no activity or no people). Macro-movements involved jumping, squatting, and spinning. On the other hand, micro-movements comprised waving, punching, push-pull motions, clapping, and rubbing hands. For each activity and device pair, approximately 1200 samples were collected. This resulted in a total of 12,095 samples for each device pair across all activities. Considering the two device pairs used in the experiment, a total of 24,190 samples were collected. Table 6 provides a detailed breakdown of the number of samples collected for each activity using a single device pair. Each activity is performed within a 5-s window. Our dataset focuses on capturing the core aspects of activities, encompassing both macro-movements like walking and sitting, as well as more subtle micro-movements such as hand gestures. While some activities may involve gradual transitions between these core movements, the essence of each movement itself can be effectively captured within a 5-s window. This duration allows us to focus on the key aspects of the activity while maintaining manageable data collection times. Itʼs important to consider the trade-off involved. Capturing continuous movements exceeding 5 s would undoubtedly provide a more comprehensive picture. However, such an approach becomes increasingly challenging and time-consuming during data collection. Striking a balance between capturing essential details and ensuring data collection feasibility is crucial. A 5-s window allows us to collect a larger volume of data encompassing a wider range of activities within a reasonable timeframe.

**Table 6:** Number of samples collected for one pair of ESP32-S3 devices for 10 human activities.

Number of samples collected for each activity									
Baseline		Micro-movement					Macro-movement		
No activity (No people)	Standing	Waving	Punch	Push pull	Clap	Rub hand	Jump	Squat	Twist
1201	1204	1210	1218	1216	1210	1240	1224	1202	1170

It's important to note that the final number of usable samples per activity within the dataset varies between 1170 and 1240 (as shown in Table 6). This variation arises from the data cleaning process, which meticulously eliminated outliers, erroneous data points, and inconsistencies to ensure the usability, integrity and reliability of the dataset. While we initially aimed to collect a fixed sample size of 1250 for each activity, the data cleaning process identified a subset of samples requiring exclusion, resulting in varying sample sizes depicted in Table 6.

### 4.2 Experimental devices and software

**Fig. 4:**
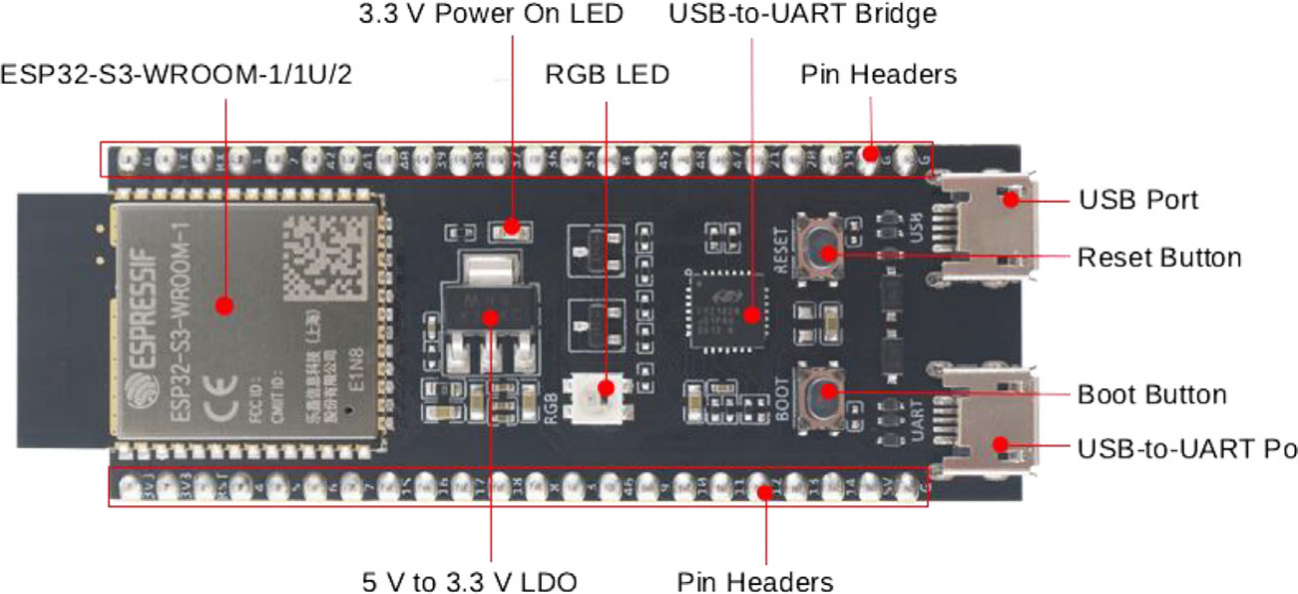
ESP32-S3-DevKitC-1 development board with key components labeled adapted from Espressif systems.

The experiment utilized two pairs of ESP32-S3-DevKitC-1 devices for collecting CSI data from both frontal (north to south) and side (east to west) orientations, setting up a diagonal network topology. Fig. 4 illustrates the device layout with labels corresponding to the official manufacturer's (Espressif) specifications [9]. Both device pairs operated on Channel 11 within the 2.4 GHz band, utilizing a 40 MHz bandwidth. The system captured data across all 166 subcarriers available on the selected channel.

**Fig. 5:**
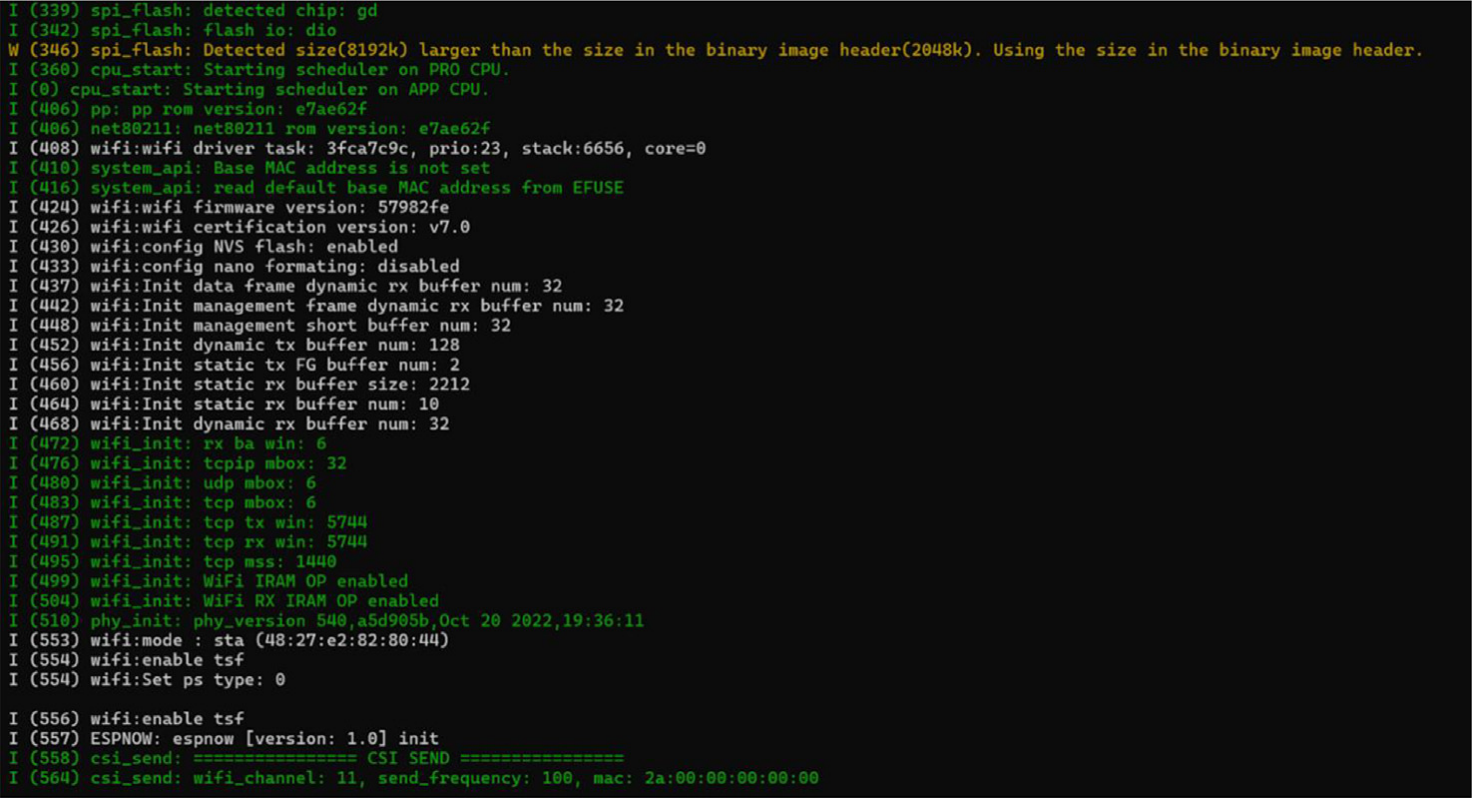
Terminal window showcasing ESP32 boot sequence, Wi-Fi module initialization and potential connection attempt.

The ESP32 devices are connected to a host computer for configuration purposes via a USB-to-Micro cable attached to the deviceʼs designated UART port. The Espressif IDF framework version 5.0 (esp-idf-v5.0.) serves as the primary platform for configuring the devices. To distinguish data collected from different orientations (front and side) to enable spatial diversity, unique MAC addresses are assigned to each device pair. The front-facing pair utilizes the MAC address 1a:00:00:00:00:00, while the side-facing pair employs 2a:00:00:00:00:00. A screenshot of the software used to enable the transmission is shown in Fig. 5.

### 4.3 Data collection environment

The data collection process was conducted within a controlled laboratory environment at Monash University Malaysia. The laboratory dimensions are specified (5 m × 8 m × 3 m). Four tripods were positioned within the laboratory, each mounting a data collection device (ESP32-S3) positioned at the height of 1.5 m. To facilitate diverse signal paths for further analysis, a diagonal network is established with transmitters positioned at the north and east corners sending signals to receivers located at the south and west corners. The experimental setup positions transmitting and receiving ESP32-S3 devices 1.5 m apart, forming a network around participants at the center who perform various activities.

Both sender devices were configured and powered using external power banks. The receiver devices were connected to the researcher's laptop via 3-meter USB-to-Micro cables for data reception. To minimize distractions and potential interference during data collection, the environment was solely occupied by the participant performing the designated activities. Additionally, to ensure a controlled data collection process, no personal electronic devices such as smartphones, smartwatches, or keys were permitted within the laboratory during data acquisition. To account for potential variations in environmental noise and interference, data collection was conducted across different time periods, including periods known for higher activity levels.

**Fig. 6:**
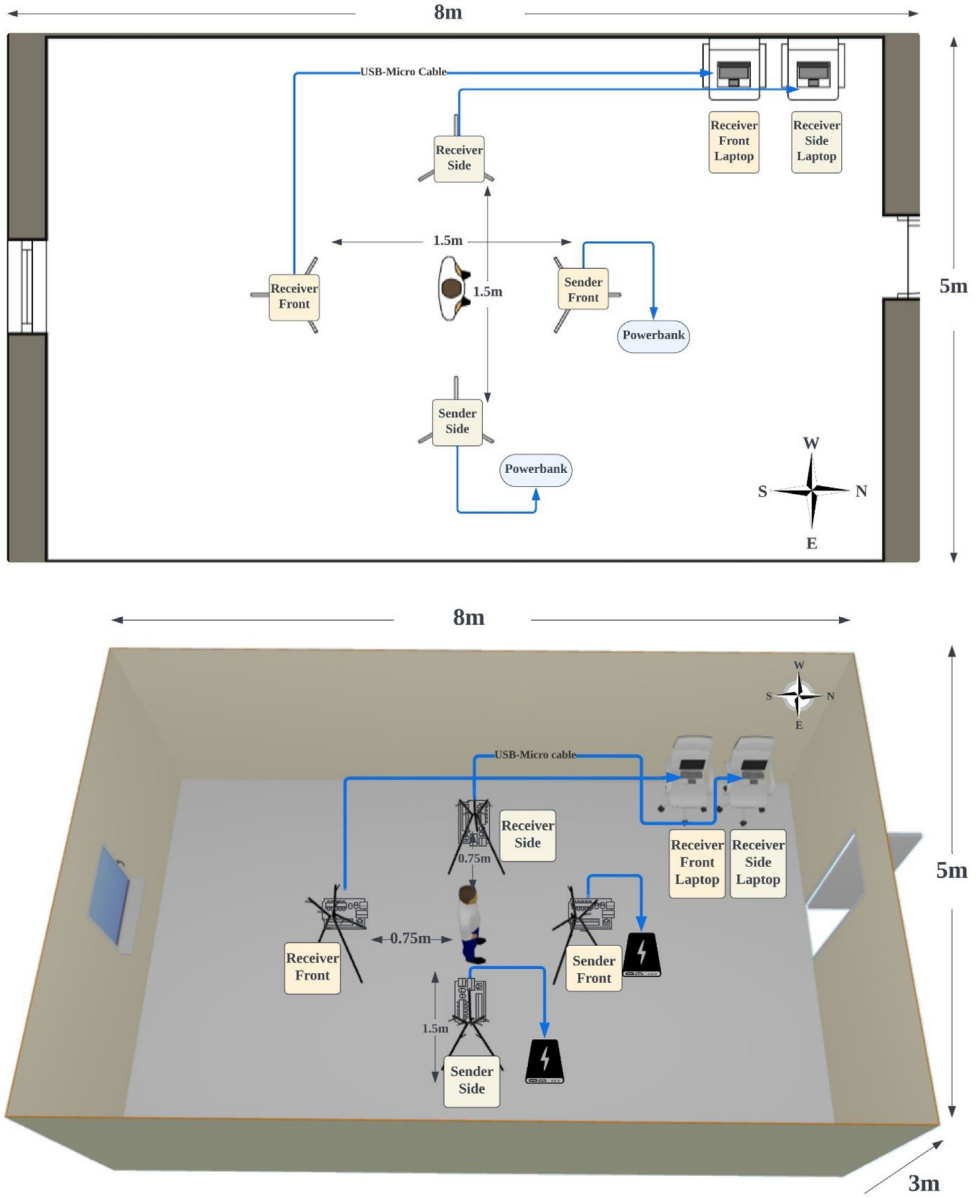
Laboratory Setup for CSI Data Collection with ESP32 Devices: 2D and 3D Layouts.

The laboratory testbed layout is illustrated in both 2D and 3D in Fig. 6. Four ESP32-S3 development boards are mounted on tripods, positioned at the cardinal points (north, east, south, and west) surrounding a central marked area. A participant would stand in this central area to perform designated activities while CSI data is collected from the ESP32 receiving devices via host computers.

### 4.4 Experimental procedure

Participants were instructed to perform designated activities within a 5-second interval while standing in the center of the four tripods. To initiate data collection, a designated researcher served as an assistant, using pre-configured commands on both receiver laptops. These commands specified the number of collection intervals, with each interval corresponding to 5 s, and the filename format for the comma-separated value (CSV) files (activity_X.csv, where X represents the sample number). The assistant then initiated data collection on both receiver devices simultaneously.

**Fig. 7:**
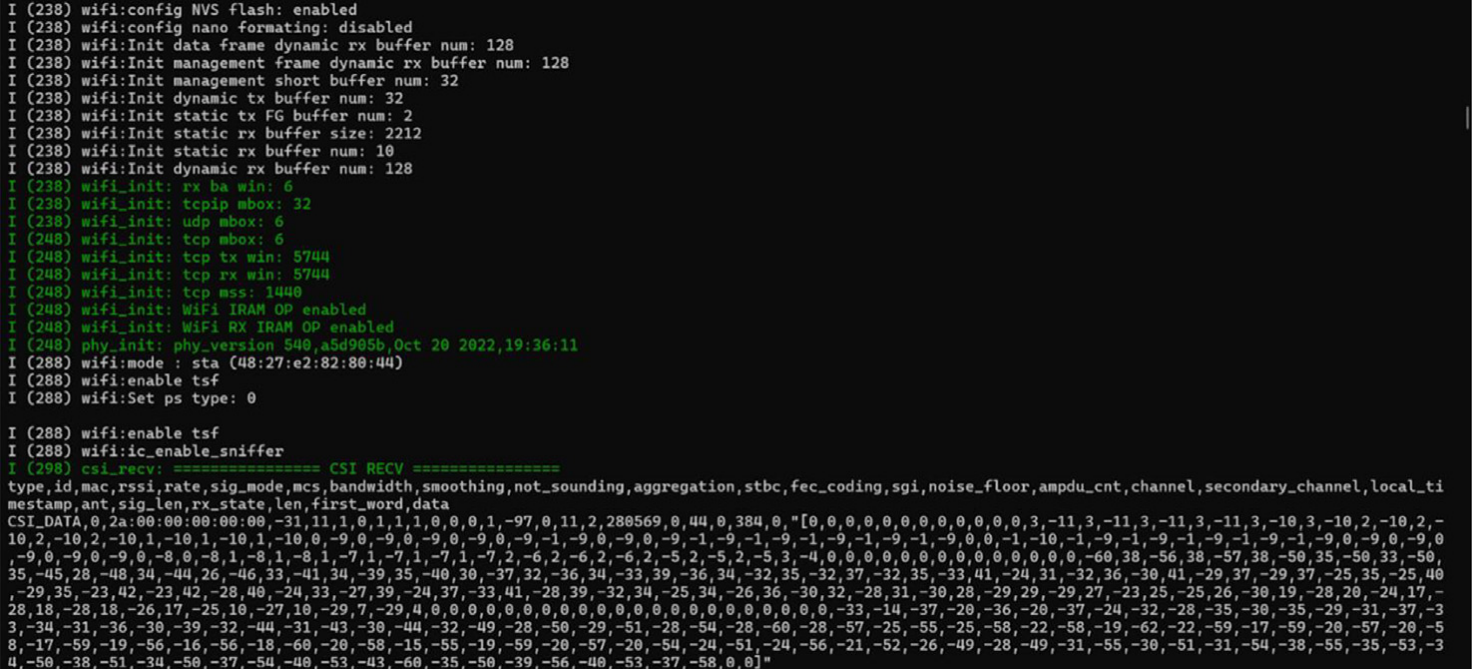
CSI data acquisition process from ESP32 devices.

Data collection proceeded continuously for 21 intervals, resulting in a total duration of 105 s. During each interval, the system was expected to capture approximately 20 data samples for the designated activity. The data file corresponding to the first interval (5 s) was subsequently removed, as the research assistant was still positioned within the laboratory space during this initial period. Following acquisition by receiving ESP32 devices, CSI data undergoes transmission to host computers for subsequent analysis in numerical formats as depicted in Fig. 7. The data is ultimately archived in CSV format, facilitating convenient access and utilization for SHD-HAR dataset construction.

### 4.5 Data annotation

Data Format Specification: The dataset stores CSI measurements for 10 activities as numerical values in CSV files. Each activity is represented by a matrix with dimensions ranging from 300 to 450 × 25, where rows represent the number of packets and columns represent different information extracted from ESP32 as listed in Table 2. For each activity, the data is collected within a continuous 5-second window, which can be captured by 300–450 discrete packets. There are two CSI measurements for each activity at the same time, one from the frontal and side orientations. Annotation Platform: The annotation process is automated using scripting where the acquired data from one pair of ESP32 device is automatically stored in a CSV file with all labels on the top row and left-most column.

Annotation Synchronization: The CSI data is synchronized with the signals transmitted by the ESP32 devices within a defined 5-second window. This synchronization ensures that the captured CSI measurements directly correspond to the activity being performed. During this 5-second window, participants repeatedly perform a specific activity for a duration ranging from 3 to 5 s. This allows for capturing variations within an activity execution while maintaining a consistent data collection timeframe. Additionally, the number of packets transmitted by the ESP32 devices within the 5-second window is not fixed, but typically falls within the range of 300–450 packets.

Activity Segmentation: The dataset captures each activity for 5 s, considered as a single sample. To account for variations within activities, each sample is repeated 1250 times, resulting in 1250 samples per activity. Additionally, data collection considers both frontal and side orientations of the participant, effectively doubling the samples to 2500 per activity (1250 for each orientation). For clear identification, each sample is labeled using the format “ActivityX.csv,” where X represents a unique identifier ranging from 1 to 1300, corresponding to the specific repetition within an activity and orientation.

Data Processing and Usability: Our initial goal is to acquire 1250 samples per activity. However, during the data preprocessing stage, a data quality assessment identified a subset of samples exhibiting potential errors, outlier or inconsistencies. To ensure the integrity and reliability of the dataset, these samples were excluded from further analysis. This data cleaning process resulted in a final usable sample count per activity ranging from 1170 to 1240. A detailed breakdown of the final sample count for each activity can be found in Table 6.

Data Labelling: Each file within the dataset adheres to a consistent labeling scheme that directly links the sample to the corresponding activity performed during the 5-s window. Table 6 provides a detailed list of the ten recorded activities. The filename format reflects this scheme, with “ActivityX.csv” representing the Xth sample for a specific activity. For example, “clap101.csv” signifies the 101st sample collected during the clapping activity. This labeling approach facilitates efficient identification and retrieval of activity-specific data for further analysis. The preprocessed and labeled samples are organized within a root folder named “raw.” This root folder contains two subfolders: “front” and “side.” These subfolders correspond to the participant's orientation during data collection (frontal or side). Each subfolder further branches into ten subfolders, named according to their respective activities as listed in Table 6. Within each activity subfolder, the final, usable samples range from 1170 to 1240, ensuring a consistent and well-structured dataset for future research endeavors.

Data Conversion and Labelling: Since HAR research primarily relies on CSI amplitudes for training deep learning models, our dataset offers a convenient data extraction method. This method retrieves the CSI amplitudes from the preprocessed raw data, creating a separate amplitude data file for each activity. Each sample in the raw data is transformed into a matrix with dimensions ranging from 300 to 450 × 166. The 166 columns represent the number of subcarriers used for signal transmission. These amplitude matrices are then stored in new “ActivityX.csv” within a dedicated “amplitude” folder. The “amplitude” folder resides at the same level as the “raw” folder, fostering a clear and accessible directory structure for researchers. The subfolder structure within the “amplitude” folder mirrors the organization of the “raw” folder. This consistency simplifies navigation and ensures researchers can readily locate the corresponding amplitude data for each activity.

## Limitations

The data described in this article for HAR has some limitations that restrict how well models trained on it perform in real-world situations.

While the controlled environment (5 m × 8 m × 3 m) offers advantages like clean and consistent data, it also introduces limitations in capturing the full spectrum of real-world scenarios. The limited space restricts the range of movements and object interactions the data encompasses. This could lead to models struggling with activities that involve larger spaces or more intricate interactions with objects.

Additionally, the controlled environment lacks the diversity of real-world settings. Factors like the presence of multiple people, various furniture arrangements, and diverse materials (carpets, rugs, different wall textures) are all absent. These missing elements can potentially hinder the generalizability of models trained on this dataset, as they might not be equipped to handle the complexities encountered in real-world applications. Our dataset employs 5-s snapshots to capture activities. This approach offers advantages in terms of data collection efficiency and focusing on core movements. However, it also introduces limitations when considering the full complexity of real-world activities. One key limitation lies in capturing activities with gradual transitions or preparatory phases that extend beyond 5 s. This limited window can hinder model generalizability, as models trained on the dataset might struggle to recognize activities in real-world scenarios where these variations are present. Furthermore, activities with extended durations can also be impacted. Walking across a large room, for example, might only be partially captured within a 5-s window. This lack of exposure to the full range of movement patterns could lead to models struggling with activities that last longer than the chosen timeframe. The focus on core movements within the 5-s window, while beneficial for data collection and analysis, comes at the expense of capturing the richness and context of real-world activity variations. Activities with intricate preparatory phases, extended durations, or complex transitions might not be fully represented in the dataset. This can limit its applicability for tasks requiring recognition of such activities. The lack of participants with more extreme body types introduces a limitation in the datasetʼs ability to comprehensively explore the impact of weight and height on CSI-based HAR. Models trained on this data might struggle to generalize accurately to scenarios involving individuals with significantly different body compositions.

Fixed device positions limit data on how people affect CSI measurements. Since the devices are static during collection, the data might not capture how people moving around the environment alter signals. This can make models struggle to adapt to the dynamic changes caused by movement.

Single-person focus restricts real-world applicability. The data only captures single-person activities. Real-world environments often involve multiple people interacting, and this dataset might not provide enough information for models to handle these multi-person scenarios effectively.

## Ethics Statement

Informed written consent was obtained from all participants, and the data collection was carried out following the procedures approved by Monash University Human Research Ethics Committee (MUHREC) in compliance with the guidelines set out in the Australian ‘National Statement on Ethical Conduct in Human Research.’

## CRediT authorship contribution statement

**Wei Ern Wong:** Conceptualization, Methodology, Software, Data curation, Writing – original draft, Visualization, Investigation. **An Hong Wong:** Conceptualization, Methodology, Software, Data curation, Writing – original draft, Visualization, Investigation. **Wei Qi Peh:** Conceptualization, Methodology, Software, Data curation, Writing – original draft, Visualization, Investigation. **Chee Keong Tan:** Supervision, Software, Validation, Writing – review & editing.

## Data Availability

Spatially-diverse High-dimensional Channel State Information (CSI) based Dataset for Human Activity Recognition (HAR) (Original data) (Zenodo).
